# Test characteristics of the tuberculin skin test and post-mortem examination for bovine tuberculosis diagnosis in cattle in Northern Ireland estimated by Bayesian latent class analysis with adjustments for covariates

**DOI:** 10.1017/S0950268819000888

**Published:** 2019-05-29

**Authors:** M. J. H. O'Hagan, H. Ni, F. D. Menzies, A. V. Pascual-Linaza, A. Georgaki, J. A. Stegeman

**Affiliations:** 1 Veterinary Epidemiology Unit, Department of Agriculture, Environment and Rural Affairs, Upper Newtownards Road, Belfast, Northern Ireland, UK; 2 Department of Methodology and Statistics, Faculty of Social and Behavioural Sciences, Utrecht University, Padualaan, Utrecht, The Netherlands; 3 Department of Farm Animal Health, Faculty of Veterinary Medicine, Utrecht University, Yalelaan, Utrecht, The Netherlands; 4 Surveillance and Antimicrobial Resistance Branch, Department of Agriculture, Environment and Rural Affairs, Upper Newtownards Road, Belfast, Northern Ireland, UK

**Keywords:** Bayesian analysis, cattle, latent class model, *Mycobacterium bovis*, post-mortem examination, skin test, test characteristics

## Abstract

The single intradermal comparative cervical tuberculin (SICCT) test and post-mortem examination are the main diagnostic tools for bovine tuberculosis (bTB) in cattle in the British Isles. Latent class modelling is often used to estimate the bTB test characteristics due to the absence of a gold standard. However, the reported sensitivity of especially the SICCT test has shown a lot of variation. We applied both the Hui–Walter latent class model under the Bayesian framework and the Bayesian model specified at the animal level, including various risk factors as predictors, to estimate the SICCT test and post-mortem test characteristics. Data were collected from all cattle slaughtered in abattoirs in Northern Ireland in 2015. Both models showed comparable posterior median estimation for the sensitivity of the SICCT test (88.61% and 90.56%, respectively) using standard interpretation and for post-mortem examination (53.65% and 53.79%, respectively). Both models showed almost identical posterior median estimates for the specificity (99.99% *vs.* 99.80% for SICCT test at standard interpretation and 99.66% *vs.* 99.86% for post-mortem examination). The animal-level model showed slightly narrower posterior 95% credible intervals. Notably, this study was carried out in slaughtered cattle which may not be representative for the general cattle population.

## Introduction

Bovine tuberculosis (bTB) is a chronic, infectious disease caused by *Mycobacterium bovis* that affects cattle and many other mammals including humans worldwide. Infection with this bacterium often remains subclinical for a long period whilst cattle can be infectious. Diagnostics therefore must focus on effective detection of cattle at an early stage of infection [1].

The single intradermal comparative cervical tuberculin (SICCT) test, based on the detection of a cell-mediated immune response, is the main ante-mortem diagnostic tool for bTB in cattle in the British Isles [2]. Animals can be classified as reactors to the SICCT test on standard, severe or super-severe interpretation based on the cut-off point used of the measured response to the injected bovine and avian tuberculins into the skin of the neck (the test is carried out as defined within the EU Council Directive 64/432/EEC, Annex B). In 2015, standard interpretation was where the thickness at the site of injection of the bovine antigen was generally greater than the site of injection of the avian antigen by more than 4 mm. A severe interpretation was generally one in which the bovine bias was 3–4 mm. Super severe interpretation refers to animals considered positive to the SICCT test having a bovine bias <3 mm. Lowering the cut-off point will increase the sensitivity but in return decrease the specificity of the SICCT test and *vice versa* [3].

In Northern Ireland, all cattle over 6 weeks old are tested on at least an annual basis and positive cattle (reactors) are slaughtered followed by post-mortem examination and laboratory tests [4]. In order to confirm bTB by laboratory tests, most SICCT test reactors with visible lesions (43–60% of reactors animals in Northern Ireland [4]) are subjected to histological examination. Furthermore, those samples that show no histological evidence of bTB are subjected to bacteriological culture as are samples from a proportion of SICCT test reactors without visible lesions [5]. The SICCT test is supplemented by routine abattoir surveillance of cattle slaughtered aiming to find visible bTB lesions. Due to factors such as the microscopic size of early lesions and the time required to develop a detectable immune response, neither the post-mortem examination nor the SICCT test can be expected to detect every bTB-infected animal. Furthermore, false-negative and false-positive reactions to the SICCT test can occur due to a variety of reasons relating to both animal- and test-related factors including desensitisation, drugs, physiological status, tuberculin used, incorrect testing technique [1] and concurrent infection [6].

The sensitivity of the SICCT test reported in the literature shows a lot of variation and was reported in previous research based on summary values of field trials [1] to be between 52.0% and 100% with median values of 80.0% and 93.5% for standard and severe interpretations, respectively. Research based on meta-analyses in a systematic review of the scientific literature using Bayesian logistic regression models concluded the median sensitivity for the SICCT test (standard interpretation) to be 50% with wide Bayesian credible intervals (CrI) (95% CrI 26–78% (median sensitivity of 63% (95% CrI 40–84%) at severe interpretation) [7]. The same study stated the median sensitivity of routine post-mortem examination at meat inspection to be 71% (95% CrI 37–92%).

The specificity of the SICCT test has previously been estimated at over 99.9% [1]. Similar figures were quoted (specificity of 99.98% (95% confidence interval (CI) ±0.004%)) for standard interpretation and for severe interpretation (99.91% (95% CI ±0.013%)) [3]. The previously mentioned study using meta-analyses [7] found a median specificity for the SICCT test of 100% (95% CrI 99–100%) and a similar figure for the median specificity of routine post-mortem examination (100%; 95% CrI 99–100%).

One of the main problems in relation to determining test characteristics and true disease status is the absence of a gold standard test for bTB. Sensitivity and specificity can be estimated in such cases by using latent class models applying two or more tests to two or more populations with distinct prevalences [8]. However, this approach summarises the test results to the (sub)population level, and it is difficult to include additional evidence available from data in the analysis. The Bayesian latent class model specified at the animal level [9] offers the possibility of including animal-level information such as disease risk factors for the estimation of test characteristics.

Therefore, although latent class analyses for test characteristic estimation has been conducted previously for bTB diagnostics [10–13], the current study is novel as it aims to address the variation in test characteristic estimates by adding a range of animal-level covariates to a Bayesian model in order to provide more precise estimates of the test characteristics for the SICCT test and bTB post-mortem surveillance.

## Material and methods

An observational study encompassing all cattle slaughtered in abattoirs in Northern Ireland in 2015 was conducted. Cattle that were slaughtered but had a presenting herd from outside Northern Ireland were excluded from the analyses.

### Data collection and definition of variables

All data were extracted from the Animal and Public Health Information System (APHIS) of the Department of Agriculture, Environment and Rural Affairs (DAERA). Details on all individual cattle, cattle movements and bTB tests conducted since 1988 are stored in this database [14]. Datasets were manipulated using Microsoft Access™ (Microsoft Corporation, USA) and subsequently analysed using R version 3.2.3 (The R Foundation for Statistical Computing) and JAGS version 4.1.0 (http://citeseerx.ist.psu.edu/viewdoc/summary?doi=10.1.1.13.3406).

Data included in the analyses were based on information at animal level and test level. The data presented at animal level included individual measures on breed, sex, age at death, days from last SICCT test to slaughter and last SICCT test reason. The days from the last SICCT test was included in order to take account of animals with their last SICCT test being negative becoming infected before being slaughtered (i.e. to account for the fact that the two tests (SICCT and post-mortem) are non-contemporaneous). Breeds were categorised as breeds mainly kept for milk production (dairy) and non-dairy breeds. In relation to sex, three categories were constructed: female, non-castrated male (bull) and castrated male (bullock). Age at death was entered into the model as a continuous variable. The last SICCT test reason (i.e. the reason for the last SICCT test being conducted prior to slaughter) was divided into three categories; i.e. routine (in situations where no risk of bTB infection was suspected to be in the herd), at risk (in situations where the herd/animal was at increased risk of having bTB infection) and restricted (in situations where SICCT test reactors or animals with lesions at routine slaughter were found or the herd was at high risk of having bTB infection) [5]. The duration in days from the last SICCT test to slaughter was not included in the final animal-level model as reasoning from a biological perspective suggests that the fact that the two tests are non-contemporaneous should not matter in the case of bTB. It is estimated that the time period from the point of infection to reactivity to the SICCT test is approximately 2–3 weeks [15]. Thereafter bTB develops into a chronic infection with the formation of granulomata with variation in the immune responses over time (based on intermittent flare ups caused by the dynamics between the infection and body's immune system) followed by the animal having a lifelong infection compared to a very small window of the incubation period where detection would be missed. Furthermore, the minimum SICCT testing interval in Northern Ireland is 2 months with the median being much higher over the population being monitored. Animals also have to be at least 18 months before they are slaughtered (unless they are found to be SICTT reactor before that). In order to check the validity of this reasoning, we ran the animal-level model in two ways: (1) based only on cattle that had ⩽45 days from the last SICCT test to slaughter; (2) based only on cattle that had ⩽23 days from the last SICCT test to slaughter.

The data presented at test level were based on the test-related information of the last SICCT test the animal was subjected to prior to slaughter and the tests after slaughter (including the post-mortem inspection result in the abattoir, the histology test and the bacteriological culture test) [15]. The interpretation of the SICCT test was based on recorded measurements of the net bovine rise (NBR), calculated as the increase (in millimetres) at the bovine tuberculin (Lelystad) injection site greater than any increase at the avian tuberculin injection site when measured after 72 h (as per EU Council Directive 64/432/EEC, Annex B). A standard interpretation is read where the thickness at the site of injection of the bovine antigen is generally greater than the site of injection of the avian antigen by more than 4 mm. A severe interpretation is generally one in which the bovine bias is 3–4 mm [13].

Cattle in the dataset were slaughtered in one of 10 abattoirs in 2015. However, as practically all SICCT test reactors were slaughtered in one slaughter house (abattoir E), posterior estimates of test characteristics were obtained on both the entire dataset and data from animals slaughtered in abattoir E only. Background analysis of lesion distributions between abattoir E and all other abattoirs were conducted in order to assess bias in relation to post-mortem examination techniques between abattoir E and the other abattoirs.

### Data analysis

#### Hui–Walter model

**Fig. 1.**
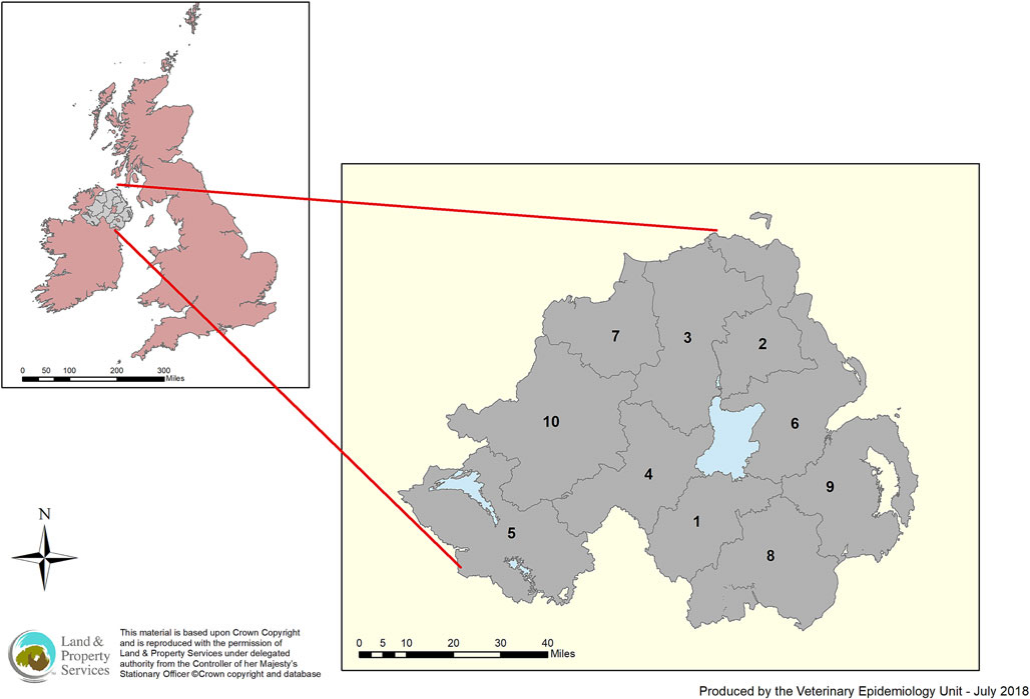
Spatial distribution of the ten Divisional Veterinary Office (DVO) areas in Northern Ireland.

The Bayesian Hui–Walter latent class model [8] was constructed to estimate the test characteristics of the SICCT test and post-mortem inspection for bTB. The 10 Divisional Veterinary Office (DVO) areas were treated as 10 subpopulations in Northern Ireland (see Fig. 1). Animals were allocated to a DVO area based on the location of the last herd they resided in before slaughter. We assumed that the 10 subpopulations submitted to slaughter had distinct proportions of bTB-infected cattle and sensitivity and specificity of the two tests were constant across populations. Based on a previous similar study [13], the two tests were assumed to be independent, conditional on the true disease status of bTB.

To check whether the risk factors had an impact on the posterior estimation of sensitivity and specificity for both tests, the Bayesian Hui–Walter model was further applied to stratified samples. Each time the entire dataset (i.e. all cattle slaughtered) was stratified into two or three samples by one of the five risk factors. The continuous covariates, age at death and days from last SICCT test to slaughter were categorised for the purpose of data stratification. Age at death was divided into two categories, i.e. ⩽2 and >2 years. This cut-off point was used as the majority of cattle bred for meat production are slaughtered by 2 years of age. The duration in days from last SICCT test to slaughter was divided into two categories, i.e. ⩽45 and >45 days. This cut-off point was chosen in line with previous research [12].

Non-informative beta prior distributions were specified for the test characteristics (i.e. sensitivity, specificity) and the true proportion of the diseased in each subpopulation. The analyses were repeated using informative priors based on the finding of previous research [7] to see whether this significantly changed the results.

The model represented the risk of being bTB positive. Sensitivity and specificity estimates were considered independent of all covariates.

#### Animal-level model

As can be seen in the Bayesian Hui–Walter approach, stratifying data by a certain risk factor made it possible for us to assess the effect of the risk factor on the estimation of test performance. However, this approach could only investigate one risk factor at a time. In addition, the continuous covariates such as age at death had to be coded into categorical variables prior to data stratification which might cause loss of information.

To estimate the test sensitivity and specificity for SICCT test and post-mortem examination while taking the possible risk factors into account, a Bayesian logistic regression model was constructed at the animal level [9]. The (latent) true bTB infection status for each animal was linked to the joint test results of the SICCT test and post-mortem inspection of each animal, expressed in the form of test sensitivity and specificity. The probability of an animal being bTB infected was the dependent variable in the logistic regression model whereas the animal-level covariates were the predictors. The advantage of this modelling method is that the effect of multiple risk factors can be assessed simultaneously, and continuous covariates can be incorporated without categorisation. In our analysis, the animal-level measures on breed, sex, age at death and last SICCT test reason were included as risk factors in the logistic regression model for animals that had ⩽45 days (or ⩽23 days) from last SICCT test to Abattoir E (see Appendix B for model code).

Non-informative normal prior distributions were specified for the regression coefficients of the risk factors. Only individual records that consisted of no empty cells from any of the variables mentioned above were used for the analysis. Backward model reduction was performed by comparing the deviance information criterion (DIC) values among the competing models.

For all analyses, four Markov Chain Monte Carlo (MCMC) chains were sampled. Within each chain, the first 5000 samples were discarded as the burn-in phase, and the subsequent 10 000 samples were used for posterior parameter estimation. Convergence was visually inspected by using trace plots.

## Results

### Descriptive results

In total, 413 383 cattle were slaughtered in abattoirs in Northern Ireland in 2015. A total of 29 839 cattle (7.2%) were dismissed from the analyses due to the fact that their presenting herd was not in Northern Ireland and a further 755 animals (0.2%) had missing values resulting in 382 789 cattle being included in the study.

### Bayesian latent class analysis

#### Hui–Walter model

The posterior medians and 95% CrI obtained for the test sensitivity and specificity of the SICCT test (standard/severe interpretation) and post-mortem examination using non-informative priors are listed in Table 1 along with the estimated proportion of disease in the subpopulations (i.e. ten DVO areas). When the standard interpretation was used for the SICCT test, the estimated sensitivity (%) for the SICCT test was 88.61 (95% CrI 85.39–92.23) and the estimated sensitivity for the post-mortem examination was 53.65 (95% CrI 52.59–54.75). The estimated specificities (%) for both tests were very high (99.99 for the SICCT test and 99.66 for the post-mortem examination, respectively). Further, as expected, when the cut-off point was changed from the standard to severe interpretation, the sensitivity for the SICCT became higher (93.27; 95% CrI 90.15–96.55) while that for post-mortem examination fell slightly (50.87, 95% CrI 49.88–51.92). However, the specificity remained very high for both tests. The difference between the estimates using non-informative and informative priors was minimal (see Appendix A; Table S1).

**Table 1.** Posterior estimates (median and 95% Crl) for SICCT test (standard/severe interpretation of the variable *net bovine rise*), post-mortem examination characteristics and proportions of the diseased in the subpopulations (DVO areas) based on the entire dataset

	Standard interpretation (based on *net bovine rise*)	Severe interpretation (based on *net bovine rise*)
Sensitivity SICCT test (%)	88.61 (85.39–92.23)	93.27 (90.15–96.55)
Specificity SICCT test (%)	99.99 (99.97–100.00)	99.99 (99.96–100.00)
Sensitivity post-mortem (%)	53.65 (52.59–54.75)	50.87 (49.88–51.92)
Specificity post-mortem (%)	99.66 (99.60–99.71)	99.68 (99.62–99.73)
DVO 1 (%)	1.55 (1.43–1.68)	1.68 (1.56–1.81)
DVO 2 (%)	1.51 (1.34–1.69)	1.63 (1.45–1.81)
DVO 3 (%)	2.47 (2.29–2.66)	2.63 (2.45–2.81)
DVO 4 (%)	1.76 (1.63–1.89)	1.89 (1.76–2.03)
DVO 5 (%)	7.30 (6.83–7.78)	7.85 (7.38–8.34)
DVO 6 (%)	1.12 (0.99–1.27)	1.33 (1.18–1.48)
DVO 7 (%)	2.47 (2.20–2.76)	2.59 (2.33–2.88)
DVO 8 (%)	2.98 (2.79–3.16)	3.16 (2.97–3.34)
DVO 9 (%)	3.93 (3.66–4.18)	4.22 (3.97–4.49)
DVO 10 (%)	3.56 (3.34–3.77)	3.72 (3.51–3.93)

Posterior parameter estimates based on stratified samples are presented in Table 2. For the stratified sample that contained only animals that were sent to slaughter after 45 days from their last SICCT test, due to very few SICCT test reactors, the test sensitivity and specificity of the tests could not be estimated.

**Table 2.** Posterior estimates (median and 95% CrI) for the sensitivity and specificity for SICCT test (standard interpretation of the variable *net bovine rise*) and post-mortem examination derived from the stratified population based on risk factors

Stratified population	SICCT test		Post-mortem	
	Sensitivity (%)	Specificity (%)	Sensitivity (%)	Specificity (%)
Age at death				
⩽2 years	91.95 (85.92–98.57)	99.97 (99.88–100.00)	60.42 (58.40–63.07)	99.71 (99.60–99.82)
>2 years	86.75 (82.80–90.74)	99.99 (99.97–100.00)	50.29 (48.98–51.67)	99.63 (99.56–99.69)
Days from last SICCT test to slaughter				
⩽45 days	90.30 (87.84–92.84)	99.98 (99.89–100.00)	53.68 (52.64–54.82)	99.69 (99.53–99.84)
>45 days	–	–	–	–
Breed				
Dairy	89.85 (86.10–93.64)	99.99 (99.94–100.00)	47.32 (45.79–48.96)	99.70 (99.61–99.79)
Non-dairy	89.85 (84.80–94.98)	99.99 (99.94–100.00)	59.25 (57.67–61.11)	99.61 (99.55–99.68)
Sex				
Bull	93.33 (80.03–99.66)	99.95 (99.76–100.00)	30.94 (25.99–36.15)	99.66 (99.47–99.86)
Bullock	72.23 (62.39–83.65)	99.98 (99.89–100.00)	63.75 (60.31–70.89)	99.82 (99.72–99.93)
Female	90.77 (87.83–93.70)	99.99 (99.96–100.00)	52.92 (51.71–54.16)	99.57 (99.50–99.65)
Last SICCT test reason				
Routine	93.85 (84.75–99.55)	99.98 (99.91–100.00)	56.56 (53.11–61.19)	99.67 (99.62–99.74)
Restricted	84.51 (80.43–88.65)	99.99 (99.96–100.00)	49.50 (48.05–50.98)	99.67 (99.58–99.76)
Risk	93.27 (88.99–97.41)	99.96 (99.87–100.00)	59.73 (57.71–62.29)	99.60 (99.51–99.69)

#### Animal-level model

As the main interest is based on cattle that went from bTB-negative to bTB-positive status, the final model and the vast majority of SICCT test reactors (8956 out of in total 8963 (99.9%)) was sent to Abattoir E. Therefore, the animal-level model with risk factors was performed only on Abattoir E cattle that had ⩽45 days from last SICCT test to slaughter. The standard interpretation of the NBR was used for all subsequent analyses. No significant difference was found in post-mortem techniques between SICCT test reactors (abattoir E) and non-reactors (all abattoirs) regarding the number, nature and size of the lesions (see Appendix A; Table S2). Results from cattle that had ⩽23 days from the last SICCT test to slaughter showed similar posterior estimates (Appendix A; Table S3).

Table 3 presents the distribution of test results from the SICCT test and post-mortem examination from the samples stratified by the risk factors within Abattoir E. The risk factors age at death and days from last SICCT test to slaughter are shown as categorical variables to provide an overview of the test-positive and negative counts from both tests (Table 3). In the model where risk factors were incorporated, age at death remained continuous and was not coded into a categorical variable.

**Table 3.** Descriptive statistics of the relationship between risk factors and SICCT test and post-mortem examination results from Abattoir E

Covariate	SICCT test positive			Post-mortem positive		
	Yes	No	% of positives	Yes	No	% of positives
Age at death						
⩽2 years	3174	12 446	20.3	2048	13 572	13.1
>2 years	5777	36 438	13.7	3400	38 815	8.1
Days from last SICCT test to slaughter						
⩽45 days	8944	14 573	38.0	5312	18 205	22.6
>45 days	7	34 311	0.02	136	34 182	0.40
Breed						
Dairy	4063	17 736	18.6	2205	19 594	10.1
Non-dairy	4888	31 148	13.6	3243	32 793	9.0
Sex						
Bull	331	903	26.8	109	1125	8.8
Bullock	1506	18 934	7.4	1029	19 411	5.0
Female	7114	29 047	19.7	4310	31 851	11.9
Last SICCT test reason						
Routine	1219	14 300	7.9	747	14 772	4.8
Restricted	4601	20 323	18.5	2669	22 255	10.7
Risk	3131	14 261	18.0	2032	15 360	11.7

Table 4 therefore presents the final posterior parameter estimates from the best-fitting (i.e. lowest DIC) animal-level model and the effect of the risk factors on the odd ratios calculated from the regression coefficients for the risk factors. Posterior estimates from the Hui–Walter model that aggregated both test results to a cross tabulation at the DVO level for Abattoir E are also listed (Table 4).

**Table 4.** Posterior estimates (median and 95% CrI) from the Hui–Walter model and the best-fitting animal-level model with risk factors using cattle that had ⩽45 days from the last SICCT test to Abattoir E

	Hui–Walter model	
	SICCT test	Post-mortem
Sensitivity (%)	92.12 (90.90–93.33)	53.60 (52.54–54.65)
Specificity (%)	99.95 (99.71–100.00)	99.18 (98.74–99.57)
	Animal-level model with risk factors	
	SICCT test	Post-mortem
Sensitivity (%)	90.56 (89.80–91.44)	53.79 (53.00–54.69)
Specificity (%)	99.80 (99.51–100.00)	99.86 (99.55–100.00)
	Effect of risk factors (odds ratio)	
*Age at death* (per day increase)	0.99996 (0.99994–0.99998)	
Sex		
Bull	Reference category	
Bullock	0.206 (0.186–0.230)	
Female	0.596 (0.543–0.667)	
Last SICCT test reason		
Restricted	Reference category	
Routine	1.430 (1.342–1.525)	
Risk	1.804 (1.715–1.882)	

Results showed that increasing age at death was very slightly related to the decrease of the odds of bTB infection. Females and bullocks had a smaller odds of bTB infection compared to bulls. Furthermore, the odds of being disclosed with bTB was higher for animals whose last SICCT test prior to slaughter was a ‘routine’ test or a ‘risk’ test compared to animals being subjected to a ‘restricted’ test.

## Discussion

Estimation of test characteristics for diagnostic tests in the absence of a gold standard is notoriously difficult and this has been reflected by the increased use of Bayesian latent class analyses [16]. In the case of bTB diagnostics, in the absence of an accurate reference standard, these analyses have been used previously in order to estimate diagnostic test characteristics [10–13].

Even with the use of Bayesian latent class modelling, a lot of variation especially in relation to sensitivity estimates of ante- and post-mortem tests for bTB has been reported [7]. The current study aimed to apply a method to obtain more accurate estimates especially in relation to the test sensitivity by adding risk factors measured at the animal level.

By choosing two populations in order to conduct the latent class analyses, one of the issues in relation to bTB is that only cattle positive for the SICCT test will have an ‘immediate’ post-mortem result available. In order to have the availability of post-mortem results in the entire study population, it was decided to choose the study population as ‘all cattle slaughtered in 2015’. This approach means that all cattle that were recorded as reactors to the SICCT test and that were slaughtered in 2015 were in the study population but not all cattle that were negative to the SICCT test that were slaughtered in 2015. One of the consequences of this is that the prevalence estimated in the subpopulations (DVO areas – Table 1) do not reflect the prevalence in the actual DVO areas as a whole; it merely represents the bTB prevalence of all the cattle slaughtered presented by herds within these DVO areas. However, these prevalences indicate that the 10 DVO areas have distinct bTB proportions which is one of the prerequisites for Hui–Walter latent class modelling [8]. For the Bayesian logistic regression model, the distinction between populations is not required as the model is constructed on the individual animal level.

The specificity estimated for both the SICCT test and post-mortem examination was very high (>99.4%) in all analyses (i.e. all cattle slaughtered, abattoir E only, standard and severe interpretation of the SICCT test, with and without addition of animal-level risk factors) with a narrow 95% CrI. These estimates are similar to previous estimates [7] and show that both bTB diagnostic tests are very unlikely to report a false-positive animal.

The sensitivity estimates for the SICCT test (varying from 88.61% (standard interpretation) to 93.27% (severe interpretation)) were on the high end of figures reported by previous studies. The chosen population (i.e. all animals slaughtered in 2015) is potentially creating a bias for SICCT test reactors compared to SICCT test-negative animals as SICCT test reactors are always slaughtered whereas SICCT test-negative animals are not. Furthermore, previous research conducted in Northern Ireland [13] (reported sensitivity at standard interpretation 40.5–57.7%) was based on chronic bTB breakdown herds only suggesting that herds that are tested on short intervals for prolonged periods of time may lower the sensitivity of the SICCT test in such circumstances. This is confirmed to a certain degree with this study as restricted tests had a significantly lower sensitivity than risk and routine tests. Moreover, estimates for chronic bTB breakdown herds used *γ* interferon test results for their analyses creating a bias towards herds that have already been censored through the removal of SICCT test reactors during at least one previous recent herd SICCT test. The sensitivity estimates reported previously in the Republic of Ireland [10] were lower as well, whereas our estimates were more in line with previously reported figures from England [11]. This is potentially due to the difference in bTB prevalence in the cattle population [17] and the stage and details of the bTB eradication programmes showing more similarities between England and Northern Ireland than between Ireland and Northern Ireland [18]. However, it has to be noted that the current study was carried out in slaughtered cattle in Northern Ireland which do not accurately represent the general cattle population.

The estimated sensitivity of post-mortem examination was similar to previously reported figures [7]. It was noteworthy that when severe interpretation was applied, lower sensitivity for post-mortem examination was obtained (Table 1). Severe interpretation is usually applied in herds when there is already infection in the herd and therefore it has been tested recently. It follows that lesions would not have had the time to develop to the ‘visible’ stage in terms of post-mortem inspection. Conditional dependence between the SICCT test and post-mortem inspection was therefore suspected. However, the Bayesian latent class models with covariance between the two tests added were not identifiable without informative priors for the test covariance. Only when informative priors were added to the covariance parameters of the tests, convergence was reached. Furthermore, posterior estimates were sensitive to the changes of informative priors for the test covariance (results not shown). This is not in agreement with a previous similar study in Northern Ireland [13] where authors found a minimal difference in the parameter estimates between the model where conditional dependence was incorporated and the model where conditional independence was assumed among the tests. More studies are needed for the investigation of the covariance between the SICCT test and post-mortem examination. Analyses based on data for abattoir E only were conducted in order to account for potential differences in the post-mortem examination conducted between abattoir E and the other abattoirs based on the fact that abattoir E was the destination for practically all (99.9%) SICCT test reactors. Distributions of post-mortem lesions by number, nature and size showed no differences between SICCT test reactors and those detected by post-mortem inspection only. This was backed up by the current study finding no significant differences in estimated test characteristics between abattoir E and the rest of the abattoirs (results not shown).

The estimates for the sensitivity and specificity of the tests varied among the stratified samples (Table 2), indicating that the parameter estimation was affected by the risk factors. Comparing the test characteristic results between the Hui–Walter model and the animal-level model including the risk factors age at death, days from last test to slaughter, sex and last SICCT test reason showed that adding the risk factors created smaller 95% CrI (Table 1 *vs.* Table 4). This suggests that by adding animal-level risk factors, we included more information to estimate the sensitivity and specificity for the SICCT test and post-mortem examination.

Furthermore, the relationship between each risk factor and the true disease status was assessed and quantified. The model evaluated possible risk factors for the bTB infection status of the animal as detected by these two diagnostic tests within the population of cattle slaughtered in 2015. The best-fitting model indicated that the risk of having a positive bTB status was significantly influenced by the animal-level characteristics age at death, days from last SICCT test to slaughter, sex and last SICCT test reason.

Age at death was negatively correlated to the odds of bTB infection, namely animals with an older age were indicated to have slightly lower odds (0.99996). Increasing age is a risk factor for bTB breakdown [19], but similarly it is protective in relation to the development of visible lesions [5, 20]. Furthermore, compared to bulls, female and castrated male animals (bullocks) tended to have smaller odds of bTB infection detection (0.60 and 0.21, respectively). Sex was not shown to be a risk factor for bTB infection, once adjusted for age, in studies previously conducted in the Republic of Ireland [21], but SICCT test-positive bulls were shown to be less likely to develop visible lesions [5]. Relative differences in bTB disclosure in relation to sex may be masked by differences in longevity of beef and dairy cattle and different ‘between and within’ herd movements and contacts experienced [19].

The odds of bTB infection was estimated to be 1.43 times higher for animals that were subjected to a ‘routine’ test prior to slaughter than for animals that were subjected to a ‘restricted’ test (reference category), whereas the odds of bTB infection for animals subjected to a ‘risk’ test prior to slaughter was estimated to be 1.80 times higher than the animals subjected to a ‘restricted’ test. It is worth noting that 58.8% slaughtered cattle that had ⩽45 days from last test to slaughter at Abattoir E were from the ‘restricted’ farms, and 61.0% when all abattoirs were included. Furthermore, no significant relationship was found between the odds of bTB infection and the animal-level characteristic breed.

It should be noted that as our study was carried out in slaughtered cattle in Northern Ireland, the estimated effect of the risk factors on the odds of bTB infection may not be representative for the general cattle population (1.75 million cattle). Further research may adapt this model to the general population.

## Supplementary material

For supplementary material accompanying this paper visit https://doi.org/10.1017/S0950268819000888. click here to view supplementary material
