# Supplementary material for: Test characteristics of the tuberculin skin test and post-mortem examination for bovine tuberculosis diagnosis in cattle in Northern Ireland estimated by Bayesian latent class analysis with adjustments for covariates
Source: Epidemiol Infect. 2019 May 29;147:e209. doi: 10.1017/S0950268819000888 (PMC6624860; doi:10.1017/S0950268819000888)
Supplement: Supplementary file 1 [file S0950268819000888sup001.docx]

**Appendix A**

**Table S1.** Posterior estimates of the test sensitivity (Se) and specificity (Sp) for SICCT and post-mortem with non-informative priors or informative priors.

|  | SICCT test | |  | Post-mortem | |
| --- | --- | --- | --- | --- | --- |
|  | Sensitivity (%) | Specificity (%) |  | Sensitivity (%) | Specificity (%) |
|  |  | | | | |
| **Non-informative prior for Se for SICCT** | 88.61  [85.39 – 92.23] | 99.99  [99.97 – 100.00] |  | 53.65  [52.59 – 54.75] | 99.66  [99.60 – 99.71] |
|  |  |  |  |  |  |
| **Informative**  **Prior for Se for SICCT** | 87.81  [84.76 – 90.86] | 100.00  [99.97 – 100.00] |  | 53.27  [52.56 – 54.74] | 99.65  [99.62 – 99.72] |

**Table S2.** Description of number (%) of animals with lesions in lymph nodes (LN) or close proximity of a particular location, median number of sites per animal having lesions (1-9 with 9 referring to ≥ 9), nature (mode; 1=calcified; 2=purulent; 3=caseous) and size (median; 1-10 mm with 10 referring to ≥ 10mm) of the lesions for SICCT test reactors and non-reactors with positive post mortem results.

| **Location** | **SICCT positive** | | | | **SICCT negative** | | | |
| --- | --- | --- | --- | --- | --- | --- | --- | --- |
|  | **Number (%) of animals with lesions** | **Number of sites (median)** | **Nature (mode)** | **Size (median)** | **Number (%) of animals with lesions** | **Number of sites (median)** | **Nature**  **(mode)** | **Size (median)** |
| **All sites** | 5214 (100%) |  |  |  | 288 (100%) |  |  |  |
| **Bronchio mediastinal LN** | 3653 (70.06%) | 9 | 1 | 10 | 205 (71.18%) | 9 |  | 9 |
| **Head** | 1697 (32.55%) | 9 | 1 | 10 | 86 (29.86%) | 9 |  | 10 |
| **Mesenteric LN** | 370 (7.10%) | 2 | 1 | 10 | 5 (1.74%) | 2 | 3 | 3 |
| **Lungs** | 214 (4.10%) | 9 | 1 | 10 | 34 (11.81%) | 9 |  | 2 |
| **Prescapular LN** | 81 (1.55%) | 9 | 1 | 10 | 2 (0.69%) | 5.50 | 2 | 10 |
| **Liver** | 45 (0.86%) | 4 | 1 | 10 | 14 (4.86%) | 9 |  | 10 |
| **Pleura** | 43 (0.82%) | 9 | 1 | 10 | 5 (1.74%) | 9 |  | 10 |
| **Precrural LN** | 21 (0.40%) | 2 | 1 | 10 | 0 (0.00%) | - | - | - |
| **Peritoneum** | 9 (0.17%) | 9 | 1 | 10 | 3 (1.04%) | 9 | 1 | 10 |
| **Kidney** | 8 (0.15%) | 5.50 | 1 | 6.50 | 0 (0.00%) | - | - | - |
| **Ham** | 7 (0.13%) | 2 | 3 | 10 | 0 (0.00%) | - | - | - |
| **Supramammary LN** | 4 (0.08%) | 5.50 | 3 | 7.5 | 0 (0.00%) | - | - | - |
| **Popliteal LN** | 3 (0.08%) | 1 | 3 | 10 | 1 (0.35%) | 3 | 3 | 3 |
| **Forequarter** | 3 (0.06%) | 6 | 3 | 10 | 0 (0.00%) | - | - | - |
| **All gut** | 1 (0.02%) | 9 | 1 | 10 | 1 (0.35%) | 9 | 1 | 10 |
| **Spleen** | 1 (0.02%) | 1 | 1 | 4 | 0 (0.00%) | - | - | - |
| **Foreleg** | 1 (0.02%) | 1 | 1 | 10 | 0 (0.00%) | - | - | - |
| **Pelvis** | 1 (0.02%) | 9 | 1 | 10 | 0 (0.00%) | - | - | - |
| **Retropharyngeal LN** | 0 (0.00%) | - | - | - | 5 (1.74%) | 10 |  | 10 |

No lesions were found in the following locations: submaxillary LN, all pluck, diaphragm, neck, sternum, all offal, stomach, inguinal LN, skin, hind leg, tail

**Table S3.** Posterior estimates (median and 95% CrI) from the animal level model with risk factors using cattle that had ≤ 23 days from last SICCT test to Abattoir E

|  | **Animal level model with risk factors** | |  |
| --- | --- | --- | --- |
|  | SICCT test | Post-mortem | |
| Sensitivity (%) | 93.26 [91.83 – 94.71] | 54.23 [53.33 – 55.32] | |
| Specificity (%) | 99.47 [97.97 – 100.00] | 98.47 [97.42 – 99.37] | |
|  | **Effect of risk factors (odds ratio)** | | |
| *Age at death*  (per day increase) | 0.99991 [0.99989 – 0.99993] | | |
| *Sex* |  | | |
| Bull | Reference category | | |
| Bullock | 0.210 [0.185 – 0.248] | | |
| Female | 0.588 [0.522 – 0.690] | | |
| *Last SICCT test reason* |  | | |
| Restricted | Reference category | | |
| Routine | 1.794 [1.671 – 1.934] | | |
| Risk | 1.979 [1.877 – 2.093] | | |

**Appendix B**

**### Population level model ###**

model{
  
for (j in 1:nr.pops) {
  
p[j] ~ dbeta(1,1)
 
pop[j, 1:4] ~ dmulti(par[j,1:4], n[j])
par[j, 1] <- p[j]*Se1*Se2 + (1-p[j])*(1-Sp1)*(1-Sp2)
par[j, 2] <- p[j]*Se1*(1-Se2) + (1-p[j])*(1-Sp1)*Sp2
par[j, 3] <- p[j]*(1-Se1)*Se2 + (1-p[j])*Sp1*(1-Sp2)
par[j, 4] <- p[j]*(1-Se1)*(1-Se2) + (1-p[j])*Sp1*Sp2
  
}
 
## priors
Se1 ~ dbeta(1, 1)  
Sp1 ~ dbeta(1, 1) 
Se2 ~ dbeta(1, 1)  
Sp2 ~ dbeta(1, 1)
 
}

**### Best fitting model with animal level covariates ###**

data {

for (i in 1:cum.dvo10) {
ones[i] <- 1

}
}

model{

## modelling the data on animal level
for (i in 1:cum.dvo10) {

#pop[i, 1:4] ~ dmulti(par[i, 1:4], 1)
par[i, 1] <- pi[i]*Se1*Se2 + (1-pi[i])*(1-Sp1)*(1-Sp2)
par[i, 2] <- pi[i]*Se1*(1-Se2) + (1-pi[i])*(1-Sp1)*Sp2
par[i, 3] <- pi[i]*(1-Se1)*Se2 + (1-pi[i])*Sp1*(1-Sp2)
par[i, 4] <- pi[i]*(1-Se1)*(1-Se2) + (1-pi[i])*Sp1*Sp2

## Define/compute the contribution to the likelihood from the ith observation

L[i]<- equals(tests[i, 1], 1)*equals(tests[i, 2], 1)*par[i, 1]

+ equals(tests[i, 1], 1)*equals(tests[i, 2], 0)*par[i, 2]

+ equals(tests[i, 1], 0)*equals(tests[i, 2], 1)*par[i, 3]

+ equals(tests[i, 1], 0)*equals(tests[i, 2], 0)*par[i, 4]

## incorporate animal level variables
logit(pi[i]) <- beta_0 + beta_1 * death.age[i] + beta_2 * Female[i] + beta_3 * Male[i] + beta_4 * Risk[i] + beta_5 * Routine[i]

p[i] <- L[i] / 1
ones[i] ~ dbern(p[i])
}

## prior
Se1 ~  dbeta(1, 1)
Sp1 ~  dbeta(1, 1)
Se2 ~  dbeta(1, 1) 
Sp2 ~  dbeta(1, 1)
 
beta_0 ~ dnorm(0, 0.001)
beta_1 ~ dnorm(0, 0.001)
beta_2 ~ dnorm(0, 0.001)
beta_3 ~ dnorm(0, 0.001)
beta_4 ~ dnorm(0, 0.001)

beta_5 ~ dnorm(0, 0.001)


## mean of the animal level predicted probabilities for bTB per subpopulation
mean.pi1<-mean(pi[1:cum.dvo1])
mean.pi2<-mean(pi[(cum.dvo1+1):cum.dvo2])
mean.pi3<-mean(pi[(cum.dvo2+1):cum.dvo3])
mean.pi4<-mean(pi[(cum.dvo3+1):cum.dvo4])
mean.pi5<-mean(pi[(cum.dvo4+1):cum.dvo5])
mean.pi6<-mean(pi[(cum.dvo5+1):cum.dvo6])
mean.pi7<-mean(pi[(cum.dvo6+1):cum.dvo7])
mean.pi8<-mean(pi[(cum.dvo7+1):cum.dvo8])
mean.pi9<-mean(pi[(cum.dvo8+1):cum.dvo9])
mean.pi10<-mean(pi[(cum.dvo9+1):cum.dvo10])

}
